# Europäische Digitale Souveränität: Weg zum Erfolg? – Ein Bericht zur Jahrestagung CODE 2020

**DOI:** 10.1007/s12399-020-00829-2

**Published:** 2020-12-23

**Authors:** Gabi Dreo, Volker Eiseler, Nils gentschen Felde, Wolfgang Gehrke, Udo Helmbrecht, Wolfgang Hommel, Julius Zahn

**Affiliations:** Forschungsinstitut CODE, Universität der Bundeswehr München, Carl-Wery-Str. 18–22, 81739 München, Deutschland

## Europa: Digital souverän oder digitale Kolonie?

Alle zukünftigen global marktbeherrschenden Produkte und Dienstleistungen werden in der digitalen Welt, im Cyberspace, angesiedelt sein oder zumindest stark damit interagieren. Beispiele sind Robotik, Industrieautomation, autonomes Fahren, intelligente Stromnetze, Smart City und Smart Home. Die Welt wird *smarter* und die IT die Basis unserer digitalen Gesellschaft. Digitale Technologien wie Big Data, Künstliche Intelligenz, autonome Systeme und cyber-physische Systeme erzeugen und verarbeiten die dabei anfallenden riesigen Datenmengen. Europa steht heute für ein hohes Maß an Datensicherheit und Datenschutz. Die EU ist wahrscheinlich die vertrauenswürdigste Region der Welt, wenn es um diese Themen geht. Wirtschaftlich kann dies als ein bedeutender Wettbewerbsvorteil betrachtet werden, der beibehalten und ausgebaut werden muss. Doch wie digital souverän ist Europa? Was ist der europäische Weg zur digitalen Souveränität? Diese Fragen sind auch im Kontext von Datensicherheit und Datenschutz zu betrachten.

Die Diskussionen und Beiträge der Jahrestagung des Forschungsinstituts CODE (FI CODE) der Universität der Bundeswehr München (UniBw M), CODE 2020, die vom 10. bis zum 12. November 2020 als digitale Konferenz durchgeführt wurde, haben unterschiedliche Aspekte dieser Themenstellung beleuchtet.

Der erste Tag der CODE 2020 mit den High-Level Diskussionsrunden war ein Beitrag des Bundesministeriums der Verteidigung zur deutschen EU-Ratspräsidentschaft. Zitat aus dem Programm der deutschen EU-Ratspräsidentschaft^1^: „Europa muss digital souverän werden, um auch zukünftig aus eigener Kraft handlungsfähig zu bleiben.“ Doch was ist der europäische Weg?

Die Daten und digitalen Dienste werden heutzutage fast ausschließlich von US-amerikanischen sowie zunehmend chinesischen, global agierenden Unternehmen beherrscht. Wenn die Sicherheitsbehörden in Europa bei ihrer Auftragserfüllung immer stärker von Produkten und Dienstleistungen nichteuropäischer Akteure abhängig werden, bedroht das zukünftig nicht nur in einem Krisenfall die Handlungsfähigkeit des Staates.

In einer zunehmend globalisierten Welt präsentiert sich die Europäische Union als Vorreiterin ethischer Werte; dies kann jedoch nicht die digitale Souveränität ihrer Bürger*innen oder Unternehmen garantieren. Auch aktuelle Herausforderungen im Bereich des Klimaschutzes und der Gesundheit, aktuell besonders im Hinblick auf die COVID-19-Pandemie, können nur mit Hilfe vertrauenswürdiger IT gelöst werden. Die Digitalisierung ist alternativlos.

Eine strategische Ausrichtung auf den Erhalt und Aufbau essenzieller Fähigkeiten für die Handlungsfähigkeit des Staates und seiner Einrichtungen ist wichtig, um den Schutz und die Sicherheit aller Bürger*innen auch in Zukunft gewährleisten zu können. Das bedeutet nicht zwangsläufig die Notwendigkeit einer Autarkie in allen Bereichen der Gesellschaft. Sie sollte jedoch stärker forciert werden in den Bereichen, die hohe Risiken für den Staat, die Gesellschaft und die Wirtschaft bergen. Gerade dort, wo wir uns auf andere verlassen müssen oder wollen, ist dann aber zwingend eigene Kompetenz nötig, um den Einsatz dieser Methoden oder Güter prüfen und regulieren zu können.

## Schlüsseltechnologien und strategische Perspektiven der Digitalisierung

Generalleutnant Michael Vetter, Leiter der Abteilung Cyber/Informationstechnik (CIT) und Chief Information Officer im Verteidigungsministerium, eröffnete die Konferenz und betonte die Wichtigkeit eines starken Europas, das seine Bürger*innen schützen kann. Dabei steht die Stärkung der europäischen Resilienz mit dem Fokus auf digitaler Souveränität im Vordergrund. Digitale Souveränität und Cybersicherheit kann nur durch die Zusammenarbeit von unterschiedlichen Akteuren, von der Forschung, Industrie und öffentlichen Institutionen bis hin zu Start-ups, erfolgreich umgesetzt werden.

Die Präsidentin der UniBw M, Prof. Dr. Niehuss, begrüßte die 600 online zugeschalteten Teilnehmer*innen und gab einen kurzen Überblick über die aktuellen Entwicklungen im FI CODE.

Die Bundesministerin der Verteidigung, Frau Annegret Kramp-Karrenbauer, ging in ihrer Keynote auf unterschiedliche Aspekte der europäischen digitalen Souveränität ein. Dabei betonte sie u. a. die Wichtigkeit der Zusammenarbeit, der Vernetzung und des Aufbaus von digitalen Ökosystemen und nannte hierbei explizit das EU-Projekt CONCORDIA mit derzeit über 55 Partnern, koordiniert durch das FI CODE, als ein wichtiges Programm zum Aufbau eines solchen europäischen digitalen Ökosystems. Nutzung vertrauenswürdiger IT, Minimierung der Abhängigkeiten von nichteuropäischer IT, Stärkung der digitalen Resilienz, Etablierung einer europäischen technologischen Führung sind nur einige Aspekte, die die Bundesministerin in ihrer Keynote betonte.

Die Ministerin der Verteidigung der Niederlande, Ank Bijleveld-Schouten, stellte die Bedeutung eines starken und unabhängigen Europas dar. Ferner hob die Ministerin die enge Zusammenarbeit der deutsch-niederländischen Streitkräfte in der digitalen Welt hervor.

Wolfgang Ischinger, Vorsitzender der Münchner Sicherheitskonferenz, thematisierte in der Diskussion mit den Ministerinnen die Wege zur Stärkung der europäischen digitalen Souveränität und stellte insbesondere die Relevanz von gegenseitigem Vertrauen, der Charter of Trust, heraus.

Cybersicherheit als *enabler* der digitalen Souveränität stand im Fokus des zweiten Panels, moderiert durch Prof. Manfred Broy von der Technischen Universität München. Dabei diskutierten Juhan Lepassaar, Executive Director der Agentur der Europäischen Union für Cybersicherheit (ENISA), Vizeadmiral Dr. Thomas Daum, Inspekteur beim Cyber- und Informationsraum (CIR) der Bundeswehr, Ralf Wintergerst, Vorstandsvorsitzender von Giesecke+Devrient, Dr. Annegret Bendiek von der Stiftung Wissenschaft und Politik und Jeremy Jurgens vom World Economic Forum die Herausforderungen der Cybersicherheit aus unterschiedlichen Sichten wie dem Aufbau eines europäischen Datenraums, Privatsphäre, digitale Identitäten und Datenschutz.

Der erste Tag der High-Level-Diskussionen endete mit einer Paneldiskussion über zukünftige Schlüsseltechnologien als Basis der digitalen Souveränität, moderiert durch Prof. Dr. Gabi Dreo, der Leitenden Direktorin des FI CODE. Benedikt Zimmer, Staatssekretär des Verteidigungsministeriums, betonte die Wichtigkeit der Verfügbarkeit von vertrauenswürdiger IT für die Handlungsfähigkeit der Streitkräfte. Angelika Niebler, Mitglied des Europäischen Parlaments, und Jiří Šedivý, Chief Executive der European Defence Agency, stellten ihre Sichten hinsichtlich des Aufbaus und der Förderung europäischer Schlüsseltechnologien dar. Stefan Winners, Berater bei Lakestar, einem europäischen Venture Capital, erläuterte seine Sicht auf Investments in Schlüsseltechnologien und die größten Hindernisse, die dazu führen, dass Europa keine großen IT-Champions hat. Die abschließende Frage an alle Teilnehmer*innen des Panels war: „Was ist der Weg, die Roadmap, um ein digital souveränes Europa aufzubauen?“ „Kooperation, Zusammenarbeit und Vertrauen“, war die einvernehmliche Antwort.

## Wegbereiter der digitalen Souveränität

Am zweiten Tag der CODE 2020 fanden sieben parallele Workshops statt, die traditionsgemäß einen fachlich tiefgehenden, intensiven Austausch zwischen den Expert*innen zu aktuellen Themen stimulieren. Die unterschiedlichen Themen, die in den Fachworkshops eingehend vorgestellt und diskutiert wurden, verdeutlichen aber auch die zahlreichen Herausforderungen, die es auf dem Weg zu einer europäischen digitalen Souveränität zu bewältigen gilt. An den virtuellen Workshops beteiligten sich insgesamt knapp 300 Teilnehmer*innen aus Europa, auch hier ein neuer Rekord, bezogen auf die Teilnehmerzahl. Stellvertretend werden nachfolgend die Höhepunkte von zwei der diesjährigen Workshops zusammengefasst.

### Workshop Cyber Resilience of Critical Infrastructures

Im Workshop wurde die Resilienz kritischer Infrastrukturen in systemischen Verbindungen komplexer cyber-physischer Systeme besprochen. Ein besonderer Fokus lag hierbei im Bereich der Wirtschaft und unterschiedlichen Ansätzen von Unternehmen, mit neuartigen Bedrohungsszenarien der Cybersicherheit umzugehen. Thematisiert wurden auch die Auswirkungen von Angriffen auf für staatliche Sicherheit relevante Einrichtungen, insbesondere die Bundeswehr. Künstliche Intelligenz im Bereich des Internet of Things wurde als eine Möglichkeit zur Verbesserung der Cyberresilienz genannt, hier insbesondere durch Prädiktion.

Als größte Herausforderungen im Bereich Cyberresilienz wurden die folgenden Punkte festgehalten:

- Best Practice-Beispiele mit relevanten Stakeholdern zu teilen,
- den sicheren und schnellen Austausch von Daten zu ermöglichen.

### Workshop Quantum Technology

Der Schwerpunkt dieses Workshops lag auf Quantencomputing und Post-Quantum-Kryptographie (PQC). Einerseits sind Quantumcomputing und dafür verwendete Hardware ein direktes Resultat der Anwendung von quantenmechanischen Effekten. Andererseits ist PQC eine Verbesserung von bisherigen klassischen kryptographischen Methoden, welche nun potentiellen Angriffen durch Quantencomputer standhalten müssen. Die Theorie von Quantenschaltkreisen bildet die Grundlage zum Verständnis für gegenwärtig verfügbare Hardware.

Die Algorithmen von Grover und Shor aus den 1990er-Jahren sind immer noch die besten Beispiele für die Effektivität eines Quantenansatzes. Beide Methoden bringen sowohl symmetrische als auch asymmetrische Kryptographie in Gefahr, wobei die letztere in Form von ECC^2^ und RSA^3^ stärker betroffen ist. Darum läuft ein NIST^4^-Prozess zur Standardisierung von neuen Methoden zur Verbesserung von gegenwärtigen digitalen Signaturen, Schlüsselaustauschprotokollen und Kryptographie mit öffentlichen Schlüsseln.

IBM hat erst kürzlich einen anspruchsvollen Fahrplan für Geräte mit mehr als 1000 Qubits bis zum Jahr 2023 vorgelegt. Dieser Meilenstein könnte endgültig die Tür zu einer besseren Fehlerbehandlung und -korrektur eröffnen. Daher wird man wohl PQC eher früher als später anwenden müssen.

## Innovation als Voraussetzung für digitale Souveränität

Der Nachmittag des zweiten Tags der CODE 2020 war der Innovationstagung zum Themengebiet Cyber und Informationstechnologie gewidmet, die nach ihrer Einführung 2018 nunmehr zum dritten Mal stattfand. Bernd Schlömer vom BMVg-Referat CIT I 2, das für Forschung und Technologie sowie Innovationsmanagement Cyber/IT zuständig ist, erläuterte in seiner Rolle als Juryvorsitzender einführend die Zielsetzung, für die Bundeswehr relevante technische Neuerungen aus akademischer und industrieller Forschung und Entwicklung in einem kompetitiven Verfahren zu identifizieren und die Innovator*innen sowie Bedarfsträger*innen miteinander zu vernetzen.

In diesem Jahr wurden aus mehr als 30 Einreichungen zwölf zur Präsentation im Rahmen von Pitches auf eine Dauer von maximal sieben Minuten begrenzten Kurzvorträgen ausgewählt. Auf Basis der Kurzvorträge und Diskussionen wurden die drei besten der zwölf Beiträge, die allesamt ihre Relevanz unter Beweis gestellt haben, bestimmt.

Trotz der Individualität jedes Beitrags ist erkennbar, dass in diesem Jahr der Einsatz von *machine learning*, unter anderem zur Identifikation von Fake News und zur Entscheidungsunterstützung, die fein granulierte Segmentierung von Datennetzen zur Platzierung technischer Sicherheitsmaßnahmen und die Auswertung im Internet frei zugänglicher Daten im Sinne der Open Source Intelligence Schwerpunkte der vorgestellten Innovationen darstellten.

Über den dritten Platz freute sich Tobias Appel vom Leibniz-Rechenzentrum der Bayerischen Akademie der Wissenschaften, der für seinen Beitrag zur automatisierten Erfolgsprüfung beim Einsatz von Exploits im Rahmen von Penetrationstests der eigenen IT-Infrastruktur ausgezeichnet wurde. Der zweite Platz ging an Michael Grytz von der HENSOLDT Sensors GmbH für die Vorstellung von I‑unHYDE, einem KI-basierten Werkzeug zur Aufdeckung und Analyse von Desinformationskampagnen. Den ersten Platz belegten Ingmar Heinrich und Ulf Schröter von der Rheinmetall Electronics GmbH, die einen Ansatz zur Moving Target Defence in mikrosegmentierten *Zero-Trust*-Netzen präsentierten. Alle Teilnehmer bekommen die Gelegenheit, ihre Innovationen im Nachgang zur Tagung ausgewählten Zielgruppen noch ausführlicher darzustellen.

## Digitale Souveränität erfordert digitale Kompetenzen

Erstmalig fand 2020 im Rahmen der CODE-Jahrestagung der Science Track statt, der jungen Doktorand*innen ein Forum zum wissenschaftlichen Austausch und Netzwerken bieten soll. Die Veranstaltung gliederte sich in zwei Teile, das Early Stage PhD Forum sowie das Last Stage PhD Forum. Der erste Programmpunkt bot angehenden Doktorand*innen eine Plattform, um Promotionsvorhaben bereits zu einem frühen Zeitpunkt vorstellen zu können, während der zweite Programmpunkt einen Erfahrungsaustausch zwischen weiter fortgeschrittenen Doktorand*innen mit ihren jüngeren Pendants ermöglicht hat.

Insgesamt wurden sieben Vortragende im Rahmen eines wissenschaftlichen Begutachtungsprozesses ausgewählt. Thematisch war das Programm bunt aus dem Bereich der IT-Sicherheit gemischt und rankte von sehr technischen Vorträgen auf der Ebene von Maschinenbefehlen bis hin zu semantischen Analysen sozialer Netzwerke oder Aspekten der Visualisierung im Rahmen der Ausbildung.

Wissenschaftlich begleitet wurde die Diskussion durch Prof. Dr. Aiko Pras von der Universität Twente sowie Prof. Gabi Dreo und Prof. Florian Alt vom FI CODE. Die Veranstaltung erfreute sich auf Anhieb großer Beliebtheit und konnte mit über 80 Zuhörer*innen trotz der rein virtuellen Darreichungsform einen guten Auftakterfolg erzielen. Der Science Track der CODE-Jahrestagung trägt einen zentralen Baustein zum Aufbau der wissenschaftlichen Community bei und unterstützt junge Wissenschaftler*innen auf deren Karrierepfaden.

## Footnotes

1 Auswärtiges Amt (2020). Gemeinsam. Europa wieder stark machen. Programm der deutschen EU-Ratspräsidentschaft. 1. Juli bis 31. Dezember 2020. https://www.eu2020.de/blob/2360246/d0e7b758973f0b1f56e74730bfdaf99d/pdf-programm-de-data.pdf. Zugegriffen: 03. Dez. 2020.

2 Elliptische-Kurven-Kryptographie.

3 Asymmetrischer Verschlüsselungsalgorithmus.

4 National Institute of Standards and Technology, USA.

